# Characterization of highly virulent multidrug resistant *Vibrio cholerae* isolated from a large cholera outbreak in Ghana

**DOI:** 10.1186/s13104-017-2923-z

**Published:** 2018-01-18

**Authors:** Patrick Kwame Feglo, Miriam Sewurah

**Affiliations:** Department of Clinical Microbiology, School of Medical Sciences, College of Health Sciences, Kwame Nkrumah University of Science and Technology, Kumasi, Ghana

**Keywords:** *Vibrio cholerae*, CtxAb, Tcp, Antimicrobial resistance, SXT constin, PFGE

## Abstract

**Objective:**

The purpose of this study was to investigate the virulent factors of *Vibrio cholerae* which caused an unprecedented large cholera outbreak in Ghana in 2014 and progressed into 2015, affected 28,975 people with 243 deaths.

**Results:**

The *V. cholerae* isolates were identified to be the classical *V. cholerae* 01 biotype El Tor, serotype Ogawa, responsible for the large cholera outbreak in Ghana. These El Tor strains bear CtxAB and Tcp virulent genes, making the strains highly virulent. The strains also bear SXT transmissible element coding their resistance to antibiotics, causing high proportions of the strains to be multidrug resistant, with resistant proportions of 95, 90 and 75% to trimethoprim/sulfamethoxazole, ampicillin and ceftriaxone respectively. PFGE patterns indicated that the isolates clustered together with the same pattern and showed clusters similar to strains circulating in DR Congo, Cameroun, Ivory Coast and Togo. The strains carried virulence genes which facilitated the disease causation and spread. This is the first time these virulent genes were determined on the Ghanaian *Vibrio* strains.

## Introduction

Toxigenic *Vibrio cholerae* causes an acute diarrhoeal disease called cholera, which can lead to death within hours if left untreated. Two important *Vibrio cholerae* serogroups 01 and 0139 caused periodic epidemic and pandemic outbreaks. Cholera got into Ghana in the 1970s [1, 2] and since has remained a serious public health problem in Ghana and many parts of the African continent [3]. Ghana had an unprecedented cholera outbreak in 2014, which progressed into 2015, affecting 28,975 people with 243 deaths. The disease spread to all the 10 Regions of Ghana, reaching 130 out of the 216 districts by the end of 2014 [4]. Cholera diagnosis in the 2014 outbreak in Ghana was based on “OneStep” Rapid, immunochromographic test (SD Bio-line) [5] performed on human faecal specimen, so the biotype, serogroup and serotype of the *V. cholerae* causing the infection were not known. This study determined the biotype, serogroup, Cholera toxin (CtxAB), Toxin-coregulated pilus (Tcp) genes, SXT constin (conjugable, self-transmissible, integrating element-carrying antibiotic resistance genes), fingerprint and antimicrobial susceptibility patterns of the *V. cholerae* causing the 2014 outbreak in Ghana.

## Main text

### Collection of *Vibrio* isolates

The study was conceptualized in the peak of the cholera outbreak in 2014, but was done between March and May, 2015. There was delay and the cholera outbreak ended due to difficulties in ethical clearance and laboratory logistics acquisition. Regional laboratories were targeted for *V. cholerae* isolates but these laboratories had no stored isolates. The National Public Health Laboratory (NPHRL) in Accra, stored positive rectal swabs after culture in cryo-tubes containing 20% glycerol in Tryptone soy broth (Oxoid Limited, Basingstoke, UK) at − 80 °C. These stored rectal swabs were retrieved and cultured for *V. cholerae* for this study.

### Culture of rectal swabs

Sixty-two rectal swabs taken from NPHRL were cultured on Thiosulfate-citrate-bile salts-sucrose (TCBS) agar (Oxoid Limited, Basingstoke, UK). Forty rectal swabs grew suspected *Vibrios* and single colonies were subcultured on trypticase soy agar and used for identification and characterization.

### *V. cholerae* identification tests

Growths on the trypticase soy agar (Oxoid Limited, Basingstoke, UK) tested with cytochrome oxidase. Those positive were tested with polyvalent O1 antiserum and monovalent Inaba and Ogawa antisera (Denka Seiken Co., Ltd. Tokyo, Japan). Biotyping involved (i) production of beta-haemolysis on sheep blood agar which was observed on 5% sheep blood agar after overnight incubation. (ii) Susceptibility to polymyxin B was done by placing a 50-unit polymyxin B disk on a seeded Mueller–Hinton agar (Liofilchem s.r.l. Bacteriology products, Italy) and then incubated overnight at 35 °C. The El Tor biotype is usually resistant to this concentration of polymyxin B [6] and (iii) the Voges–Proskauer test positivity was performed by adding the test organism to Voges–Proskauer broth (Oxoid, Basingstoke, United Kingdom) and incubated at 35 °C overnight. Then 0.6 mL of 5% alpha naphthol was added, followed by 0.2 mL of 40% KOH, shaken for 15 min and observed for a red colour in the top layer in the tube.

### Antimicrobial susceptibility testing of the isolates

Antimicrobial susceptibility of the isolates was determined by the Kirby-Bauer disk diffusion method on Mueller–Hinton agar (Oxoid Limited, Basingstoke, UK) with *E. coli* ATCC 25922 as control. The *V. cholerae* isolates were tested against nine antimicrobials and diameter of zones of inhibition were measured and interpreted according to the guidelines of Clinical and Laboratory Standards Institute (CLSI, 2015) (http://www.clsi.org).

### Cholera toxin gene (CtxAB) and toxin coagulated pilus (Tcp) and SXT genes determinations

DNA was extracted from the *V. cholerae* isolates using spherolyse extraction kit (Hain Lifescience, Germany). The CtxAB and Tcp primer sequences and PCR optimizations were adopted from protocols described [7] and SXT primers and PCR optimizations adopted were also described earlier [8]. All PCR primers were obtained from *Intergrated DNA Technologies* (USA). PCR was run in a reaction volume of 50 µL using the *V. cholerae* DNA template as follows:

#### Cycling conditions for cholera toxin (CtxAB)

Initial denaturation—94 °C for 3 min; denaturation—94 °C for 1 min; annealing—78 °C for 30 s; extension—72 °C for 1 min; final extension 72 °C for 10 min.

#### Cycling conditions for toxin coregulated pilus (TCP)

Initial denaturation—94 °C for 3 min; denaturation—94 °C for 1 min; annealing—59 °C for 30 s; extension—72 °C for 1 min; final extension 72 °C for 10 min.

#### Cycling conditions for SXT gene

Initial denaturation—94 °C for 3 min; denaturation—94 °C for 1 min; annealing—62 °C for 30 s; extension—72 °C for 1 min; final extension—72 °C for 10 min.

### Separation of amplicons by gel electrophoresis

The PCR products were stained with ethidium bromide and separated in 1.5% agarose gel and captured with a Kodak camera (Kodak, Japan).

### PFGE determination

The *V. Cholerae* isolates were inoculated into Carey-Blair transport medium and sent by post to the National Institute for Communicable Diseases Division, National Health Laboratory Service, South Africa, for the PFGE. In South Africa, many strains failed to grow when sub-cultured, but nine strains survived and were characterized by the Pulsed Field Gel Electrophoresis (PFGE). The strains were digested by *Sfi*I restriction enzyme and separated in 1% Seakem Gold Agarose in CHEF-DR electrophoresis system (Bio-Rad). *Salmonella* Braenderup (H9812) was used as a control strain.

### Data analysis

Data generated was analyzed with STATA StataCorp LP, USA). Categorical values were expressed as proportions and compared using Chi square tests. P value < 0.05 was considered significant.

## Results

A total of 62 rectal swabs were obtained from NPHRL-Korle Bu and Komfo-Anokye Teaching Hospital (KATH), out of which 40 *V. cholerae* were isolated. Most of the isolates being 39 (97.5%) were obtained from NPHRL, Korle-Bu, only one isolate was obtained from KATH, Kumasi. All the 40 isolates were identified to be *V. cholerae* 01 biotype El Tor, serotype Ogawa, bearing the classical CtxAB, Tcp and SXT genes where they were detected in 39 (97.5%) of the 40 *V. cholerae* isolated. One isolate from among the NPHRL strains lacked both the CtxAB and Tcp genes. SXT constin found in 39 (97.5%) of the isolates, also caused resistance to trimethoprim/sulfamethoxazole and 4 (35%) SXT constin positive isolates were resistant to chloramphenicol.

### Antimicrobial susceptibility

**Fig. 1:**
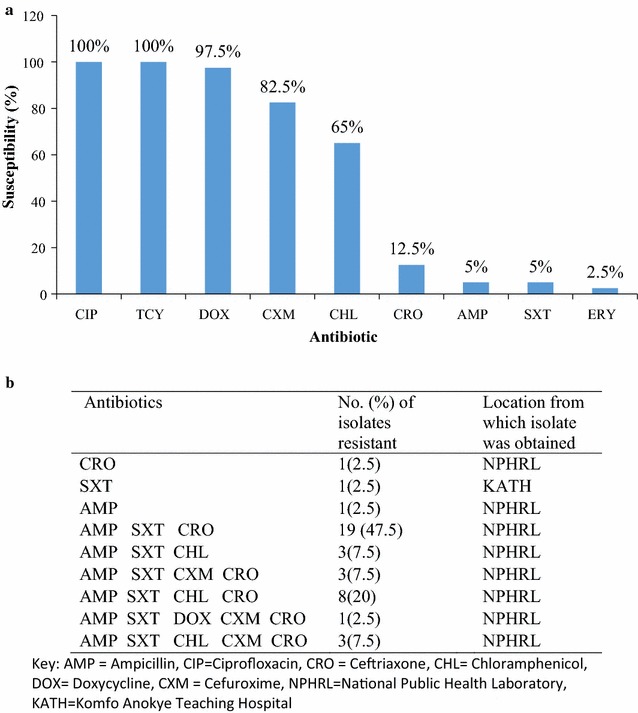
**a** Antimicrobial susceptibility pattern of Vibrio cholerae isolates of the 2014 outbreak in Ghana. **b** Multidrug resistance pattern of Vibrio cholerae isolated in Ghana

Antimicrobial susceptibility pattern of the *V. cholerae* isolates was determined. Most of the isolates (i.e. 95%), were resistant to trimethoprim/sulfamethoxazole, 90% were resistant to ampicillin and 75% were resistant to ceftriaxone. The resistance and susceptible proportions of the isolates when compared were statistically significant (P = 0.001) against ampicillin, ceftriaxone and trimethoprim/sulfamethoxazole. Eight (20%) of the isolates were resistant to ampicillin, chloramphenicol, trimethoprim/sulfamethoxazole and ceftriaxone, making the isolates multidrug resistant (resistant to ≥ 3 antibiotics). The multidrug resistance distribution of the isolates show 19 (47.5%) and 8 (20%) isolates were resistant to 3 and 4 antibiotics respectively. The resistance proportions and multidrug resistance pattern of the isolates are presented in Fig. 1.

**Fig. 2:**
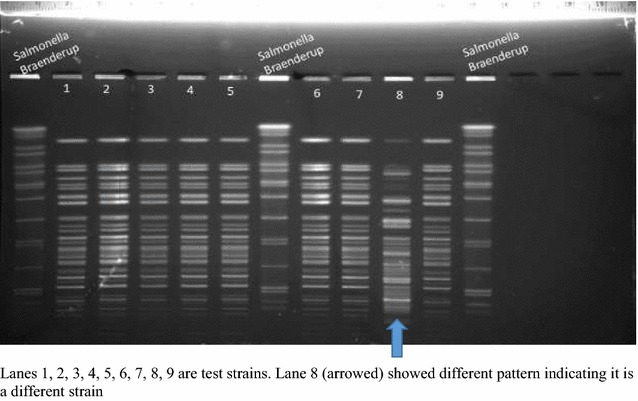
PFGE on V. cholerae O1 isolated from 2014 cholera outbreak in Ghana

**Fig. 3:**
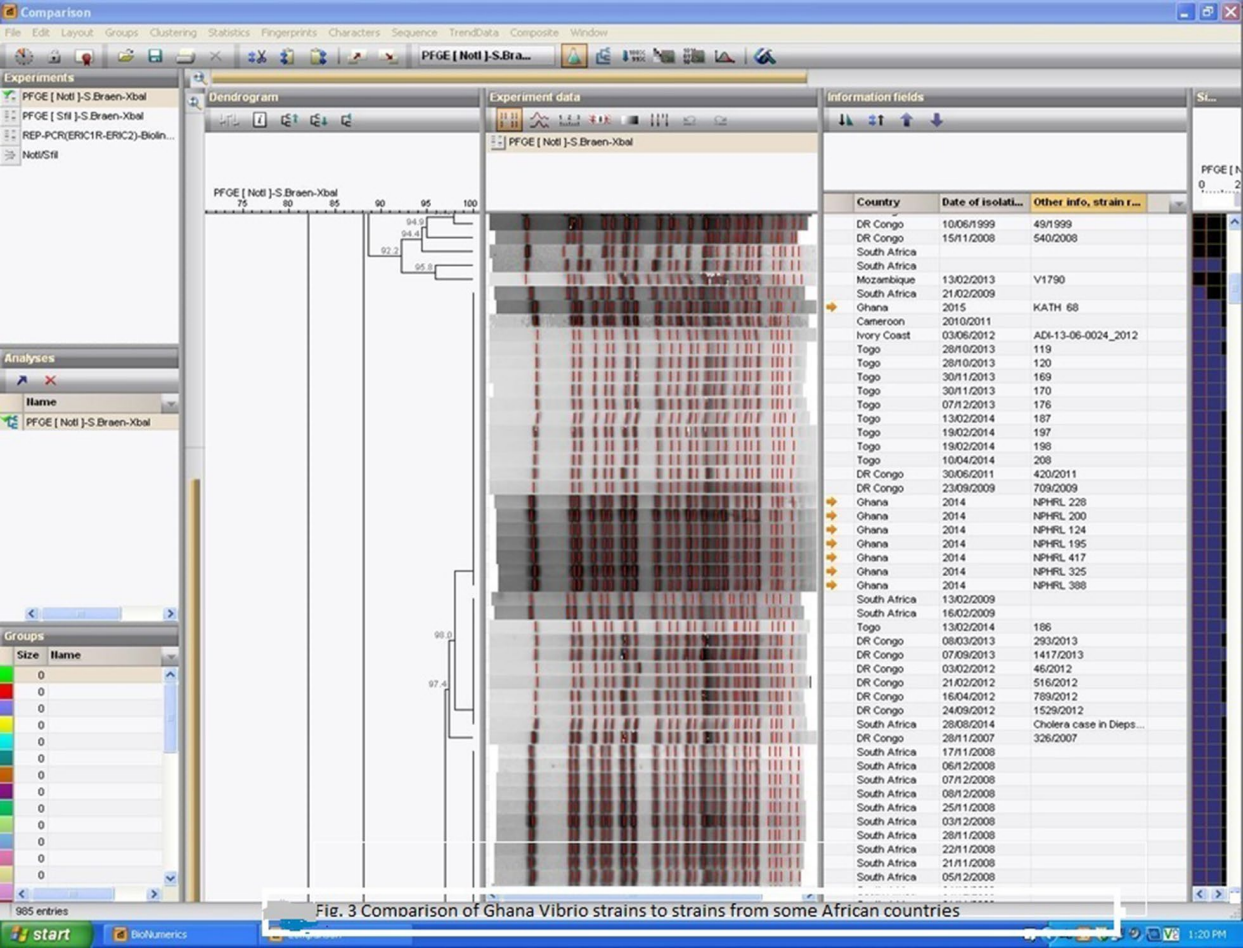
Comparison of Ghana Vibrio strains to strains from some African countries

PFGE gel produced good fingerprint patterns for eight isolates (except one). These gel patterns were captured into BioNumerics software for dendrogram analysis where they were compared with patterns of *V. cholerae* strains isolated from the African continent in the past. The gel pattern and the dendogram analysis output pattern are presented in Figs. 2 and 3 respectively.

## Discussion

In Ghana cholera is endemic and its spread is greatly enhanced by improper food hygiene, poor sanitation, environmental and waste disposal systems [9].

The cholera toxin gene (CtxAB) and toxin coregulated pillus (Tcp) gene are both needed by *V. cholerae* for its pathogenesis in humans [10], which were found in the classical O1 and the O139 serogroups initially but not the El Tor strain which caused mild diarrhoeal disease that mimicked cholera. The El Tor strains in India and Africa have now acquired the toxin genes by horizontal conjugal transfer [10] and therefore cause classical cholera. The *V. cholerae* strains isolated in the 2014 outbreak were El Tor strains but caused severe cholera and a large outbreak. The Ghana strains acquired the classical cholera toxin genes and now behaved as the classical 01/0139 biotypes [11, 12] and such El Tor biotype lineage with CtxAB genes is associated with increased virulence and increased cholera toxin production with severe disease [13]. It is unclear whether this El Tor lineage with CtxAB gene in addition to the Tcp have given the 2014 cholera strains from Ghana an increased virulence and this needs to be investigated in other studies.

Acquisition of antibiotic resistance by *V. cholerae* isolates is an important clinical phenomenon with increased risk of treatment failure and patients’ prolonged hospital stays. Though mainstay of cholera treatment is fluid and electrolyte replacement [14], antibiotic administration shortens period of diarrhoea and reduces *Vibrio* excretion in stool. Emerged *Vibrio* resistotypes have acquired SXT constin [15], which are mobilizable plasmids make many isolates multidrug resistant [16] and it is spread by open defaecation as practiced in Ghana. This study detected 95% isolates carry SXT constin with resultant resistance to ampicillin (95%) and chloramphenicol (95%). Widespread dissemination of SXT clone in the Ghanaian strains heightens growing concern over multidrug resistant *Vibrio* strains [16–18]. *V. cholerae* infection may become difficult to treat, infected individuals may harbour the organisms longer and excrete them in the environment, while *Vibrios* may serve as SXT constin reservoirs [10] for dissemination.

PFGE results in this present study show more than one strain type circulated in the cholera outbreak. *V. cholerae* are phylogenetically diverse with equally diverse virulent factors [10]. Eight Ghana strains showed similar and indistinguishable PFGE patterns, except one strain with different PFGE pattern, meaning more than one strain type caused the 2014 outbreak as seen in other studies where different clones were found in individual patients and outbreaks [19] due to phage and plasmids gain and loss. The eight strains with good PFGE patterns were committed to BioNumeric software for dendogram analysis, and were seen to share patterns previously seen in isolates from DR Congo, Togo, Cameroun and Ivory Coast.

This study reports that the 2014 cholera outbreak in Ghana was caused by *V. cholerae* biotype El Tor, O1 serogroup, serotype Ogawa, bearing CtxAB and Tcp genes and SXT genes and are similar to those already known circulating in Africa.

## Limitations

Lack of funds delayed the study, so stored samples were used, which also caused delay in shipping the isolates to South Africa leading their death, leaving only nine survivors for PFGE fingerprinting. A study involving more strains and transcriptional gene regulators such as ToxR in the Ghanaian *V. cholerae* is suggested.

## Abbreviations

CtxAB: cholera toxin encoding operon
Tcp: toxin-coregulated pilus
SXT: conjugative, self-transmissible integrating element
PFGE: pulsed field gel electrophoresis
DR: Democratic Republic of Congo
